# Genetic basis of local adaptation in the cold-tolerant mangrove *Kandelia obovata*

**DOI:** 10.3389/fpls.2024.1385210

**Published:** 2024-04-24

**Authors:** Chuangchao Zou, Yushuai Wang, Renchao Zhou, Tian Tang

**Affiliations:** State Key Laboratory of Biocontrol and Guangdong Key Laboratory of Plant Resources, School of Life Sciences, Sun Yat-sen University, Guangzhou, Guangdong, China

**Keywords:** local adaptation, population genomics, demographic history, selective sweeps, genome-environment association, mangroves

## Abstract

Understanding the genetic basis of local adaption is crucial in the context of global climate change. Mangroves, as salt-tolerant trees and shrubs in the intertidal zone of tropical and subtropical coastlines, are particularly vulnerable to climate change. *Kandelia obovata*, the most cold-tolerant mangrove species, has undergone ecological speciation from its cold-intolerant counterpart, *Kandelia candel*, with geographic separation by the South China Sea. In this study, we conducted whole-genome re-sequencing of *K. obovata* populations along China’s southeast coast, to elucidate the genetic basis responsible for mangrove local adaptation to climate. Our analysis revealed a strong population structure among the three *K. obovata* populations, with complex demographic histories involving population expansion, bottleneck, and gene flow. Genome-wide scans unveiled pronounced patterns of selective sweeps in highly differentiated regions among pairwise populations, with stronger signatures observed in the northern populations compared to the southern population. Additionally, significant genotype-environment associations for temperature-related variables were identified, while no associations were detected for precipitation. A set of 39 high-confidence candidate genes underlying local adaptation of *K. obovata* were identified, which are distinct from genes under selection detected by comparison between *K. obovata* and its cold-intolerant relative *K. candel*. These results significantly contribute to our understanding of the genetic underpinnings of local adaptation in *K. obovata* and provide valuable insights into the evolutionary processes shaping the genetic diversity of mangrove populations in response to climate change.

## 1 Introduction

Understanding the evolutionary processes and genetic basis of local adaptation has been a longstanding interest in evolutionary biology (Hoban et al., 2016; López-Goldar & Agrawal, 2021). Abiotic and biotic effects such as low and high temperature, drought, flooding, herbivore and pathogen stresses impose different selective pressures across habitats (Vanwallendael et al., 2019). The interactions among selection, gene flow and genetic drift shape genetic variation within and between populations, leading to evolutionary differentiation at different spatial scales (Orsini et al., 2013; von Takach, Penton, et al., 2021). Local adaptation can occur when genetic differentiation allows a single population to become better adapted in a particular set of environmental conditions within its range (Angert et al., 2020). The genomic architecture of natural populations thus provides an opportunity to reveal and link molecular mechanisms underlying species’ phenotypic diversity with diverse environments in which the species lives. This knowledge is important for informing management decisions in the context of rapid contemporary environmental changes (Isabel et al., 2020).

Climate adaptation is one of the most prevalent forms of local adaptation that has significant impact on the distribution of a species. Classical experimental approaches explore to which extent morphological, physiological and transcriptional responses are associated with changes in environmental factors, typically through transplants and common gardens (Carley et al., 2021; Oomen & Hutchings, 2022). Nevertheless, as most traits are polygenic, genes involved in ecological, physiological, or transcriptional changes may only be weakly related to fitness. With recent advance of sequencing technology, population genetic or genomic analyses are typically utilized to identify selective signatures linked to local adaptation, with allele frequencies expected to demonstrate ecological differentiation or correlation with specific environmental factors (von Takach, et al., 2021; Hu et al., 2022; Wang J. et al., 2022). As adaptation to climate change progresses, genetically distinct populations undergoing local adaptation may eventually become separate species if they no longer interbreed or exchange genes. Therefore, studying the genetic basis of climate adaptation can also provide insights into the evolutionary processes leading to the fixation of genetic differentiation and subsequent speciation (Levin, 2019; Dool et al., 2022).

Mangroves are a group of phylogenetically diverse woody plants that inhabit tropical and subtropical intertidal zones with great ecological and economical significance (Tomlinson, 1986; Sribianti et al., 2021). At the dynamic interface between sea and land, the structure and biodiversity of mangrove communities are sensitive to climate change (Jennerjahn et al., 2017). Mangrove forests have faced and survived several catastrophic climate change events since their origination during the Late Cretaceous-Early Tertiary period (Singh et al., 2022). Sea-level rise significantly influences the historical distribution of mangrove forests, leading to speciation in five common mangrove species via multiple cycles of mixing, isolation, and mixing (Lovelock et al., 2015; He et al., 2022). Meanwhile, low temperature stress governs the latitudinal range limits for contemporary mangrove flora (Lin, 1999) and plays a crucial role in mangrove afforestation and restoration (Ellison, 2000; Quisthoudt et al., 2012; Su et al., 2021; Chen et al., 2023). Given the ecological importance of mangroves and the potential impact of climate change on their habitats, further research into their adaptation to low temperatures is vital. The complex demographic histories of mangroves involving bottleneck, genetic drift, population expansion, and gene flow also provide a challenge for studying local adaptation.

*Kandelia obovata*, regarded as the most cold-tolerant mangrove species, is able to survive chilling temperatures as low as 4.2°C (Maxwell, 1995; Sheue et al., 2003; Zhou et al., 2007). This species can be found from the Gulf of Tonkin in the northeast to southern Japan, separated from its cold-intolerant relative, *K. candel*, by the South China Sea (Sheue et al., 2003), representing a good case in point for ecological speciation. Besides the clear differences in cold tolerance between Kandelia species, common garden studies have identified a greater cold tolerance in the northern populations of *K. obovata* compared to the southern populations (Sheue et al., 2003; Zhao et al., 2021), indicating the occurrence of local adaptation within *K. obovata*. Whole-genome bisulfite sequencing and RNA-seq of the *K. obovata* transplants suggest that modifications of DNA methylation in MADS-box genes may contribute to the adaptation to new environments, whereas the suppressed expression of lignin biosynthesis genes appears to play a role in maladaptation (Zhao et al., 2021). Additionally, the physiological and expressional analyses have highlighted several key genes and pathways that are potentially involved in cold tolerance in *K. obovata*, such as genes involved in calcium signaling, cell wall modification, and post-translational modifications of ubiquitination pathways (He et al., 2023). However, how genetic differentiation is maintained and whether the same genes underlying local adaptation to cold stress within species can also be responsible for between-species differences in cold tolerance remain largely unknown.

In this study, we investigated the genomic architecture of *K. obovata* populations along a latitudinal gradient in China using whole-genome resequencing, based on the chromosome-anchored genome assembly of *K. obovata* (Hu et al., 2020). Our analysis aimed to infer the demographic history and detect selective signatures within *K. obovata* populations, with the underlying hypothesis that local adaptation has occurred despite gene flow, driven by strong selection pressures related to temperature. We expected the signatures of local adaptation to be more pronounced in the northern population compared to the southern population. Moreover, we identified candidate genes under selection by comparing polymorphism within *K. obovata* with divergence between *K. obovata* and *K. candel*. We expected that the genes involved in local adaptation within K. obovata would differ from those under selection in the lineage of *K. obovata* since its divergence from *K. candel*.

## 2 Materials and Methods

### 2.1 Plant materials and population resequencing

We sampled leaves from a total of 46 K*. obovata* individuals, collecting plants at least 20 meters apart within each of three natural populations along the southeast coast of China (**Supplementary Table S1**). Specifically, we collected 14 individuals from Shacheng Harbor (Fuding, Fujian Province), 17 from Yanzao Village (Shenzhen, Guangdong Province), and 15 from Bamen Bay (Wenchang, Hainan Province). The fresh leaves were air-dried with silica gel before DNA extraction. Genomic DNA was then extracted using a modified CTAB protocol (Yang et al., 2008). Subsequently, DNA libraries were obtained using Nextera Mate Pair Sample Preparation Kit (Illumina USA), followed by whole-genome re-sequencing on an Illumina HiSeq 2500 platform (Illumina, San Diego, CA, USA) with paired-end reads of 150 bp (PE150). The average sequencing depth for each individual was approximately 40× (**Supplementary Table S1**).

### 2.2 Reads mapping, variant callings and SNP filtering

A high-quality chromosome-scaled assembly of the *K. obovata* genome, with a total length of 177 Mb, was published using PacBio, Illumina and Hi-C sequencing (Hu et al., 2020) and served as the reference genome for this study. Raw Illumina reads were initially processed by trimming and filtering using Trimmomatic v0.39 (Bolger et al., 2014), followed by mapping onto the *K. obovata* reference genome using Burrows-Wheeler-Alignment (BWA) v0.7.12-r1039 (Li & Durbin, 2009). Subsequently, sorted bam files were generated from sam files using SAMtools v1.6 (Li et al., 2009), and PCR duplicates were removed using MarkDuplicates in the Picard toolkit. Variants were called using HaplotypeCaller and genotyped using GenotypeGVCFs in Genome Analysis Tool Kit (GATK) (McKenna et al., 2010). The analysis solely focused on single nucleotide polymorphism sties (SNPs) and employed specific filtering criteria to reduce false positives: (1) SNPs with a read number less than three for each individual were removed; (2) SNPs with a minor allele frequency (MAF) less than 0.05 were discarded; (3) SNPs were further filtered using VariantFiltration with the following parameters: quality by depth (QD) < 2.0, Fisher strand (FS) > 60.0, mapping quality (MQ) < 40.0, mapping quality tank sum test (MQRankSum) > -12.5, and read pos rank sum test (ReadPosRankSum) < -8.0. Finally, SNP annotation was conducted based on the *K. obovata* genome using snpEff 4.3t (Cingolani et al., 2012). Following sequence alignment, removal of PCR duplicates, and SNP filtering, a set of high-quality SNPs sites were retained for the subsequent analyses.

### 2.3 Genetic diversity and population differentiation

The entire *K. obovata* genome was divided into non-overlapping 20-kb bins. Nucleotide diversity (*θ_π_*; Tajima, 1989) for each *K. obovata* population and fixation index (*F*_ST_; Weir & Cockerham, 1984) between pairwise populations were calculated within each bin using VCFtools v0.1.15 (Danecek et al., 2011). Pairwise genetic distance (D_XY_; Nei & Miller, 1990) between populations were calculated within each bin using pixy v1.2.7 (Korunes & Samuk, 2021).

### 2.4 Population structure analysis

A principal component analysis (PCA) was performed to visualize inter-individual genetic relationships using PLINK v1.90 (Purcell et al., 2007). A phylogenetic tree based on the high-quality SNPs was constructed using IQtree (Nguyen et al., 2015) with the parameter: -m GTR+F+G4+ASC, for which the input PHYLIP file was converted using vcf2phylip.py (https://doi.org/10.5281/zenodo.1257058). The number of genetic clusters (*K*) was identified using ADMIXTURE v1.3.0 with default parameters (Alexander et al., 2009). Various values of *K* ranging from 2 to 5 were tested, and the best *K* was selected based on the minimum error rate of *K* value.

### 2.5 Demographic modelling

The demographic history of *K. obovata* was inferred based on its observed population structure through the construction of a two-dimensional joint unfolded site frequency spectrum (2D-SFS) using the easySFS tools (https://github.com/isaacovercast/easySFS#easysfs), with all SNPs and the projection number equal to the individual number (i.e. 14, 17 and 15) for each of the three populations, respectively. We considered various scenarios of divergence, bottleneck, expansion, and/or migration, which were represented by different models: (i) Models 1-4: Divergence, including one-step isolation in model 1 and three two-step isolations of the *K. obovata* populations in models 2-4; (ii) Model 5-7: Bottleneck, with one population experiencing the reduction of population size and then recovery; (iii) Model 8-10: Expansion, with one population experiencing exponential population changes; (iv) Model 11-14: Migration, with asymmetric gene flow and differences in occurrence between different populations; (v) Model 15: Composite model incorporating each of the estimated best scenario for divergence (Model 1), bottleneck (Model 6), expansion (Model 10), and migration (Model 14) (**Supplementary Figure 1**).

These demographic models were compared, and demographic parameters were inferred using a coalescent simulation-based method as implemented in fastsimcoal2.6 (Excoffier et al., 2021). The initial ranges for the parameter estimation were listed in **Supplementary Table S2**. The log-likelihood for a set of demographic parameters was estimated using 100,000 coalescent simulations, with 80 conditional maximum algorithm cycles in each run and global maximum likelihood estimates obtained from 100 independent runs. The maximum likelihood value of the 100 independent runs for each model was used to compare between models using the Akaike information criterion (AIC) and Akaike’s weight of evidence tests. The model with the highest Akaike’s weight value was considered as the optimal model. The parameter confidence intervals (CIs) for the optimal model were obtained from 100 parametric bootstrap samples, independently run 100 times in each bootstrap. When converting estimates to years, it was assumed that the mutation rate and the average generation interval time in *K. obovata* were 7.86 × 10^−8^ per site per generation and 20 years per generation (average time from seed germination to seed production), respectively (He et al., 2022).

### 2.6 Detection of positive selection

Sliding window analysis was employed to detect outlier genomic regions with strong population differentiation using pairwise fixation index (*F*_ST_) and the cross-population composite likelihood ratio (XP-CLR) test (Chen et al., 2010) with a 20-kb window size and 5-kb step size. Pairwise *F*_ST_ estimates were calculated using VCFtools v0.1.15. The XP-CLR test was performed using the xpclr python module (https://github.com/hardingnj/xpclr) with default parameters. Outlier windows with both *F_ST_* and normalized XP-CLR values at least 1.96 standard deviations (SD) above the mean (one-tailed *p*-value = 0.025) were identified as the highly differentiated regions (HDRs). Sliding window analysis of Fay and Wu’s *H*-statistic (Fay & Wu, 2000) in HDRs was conducted for each population separately, in comparison with the whole genome, using ANGSD v0.921 (Korneliussen et al., 2014) with a 20-kb window size and 5-kb step size.

To detect positively selected genes, the McDonald-Kreitman (MK) test (McDonald & Kreitman, 1991) was conducted using whole-genome resequencing data for all *K. obovata* individuals obtained in this study and the reference genome of *K. candel* (He et al., 2022) as an outgroup. BlastN (Altschul et al., 1990) was used to identify homologous genes between the two Kandelia species using an e-value threshold of < 1e-10, resulting in the identification of 16,536 one-to-one orthologous genes. The number of non-synonymous (*D_n_*) and synonymous (*D_s_*) substitutions were compared to the number of non-synonymous (*P_n_*) and synonymous (*P_s_*) polymorphisms within *K. obovata* for coding sequences of each Kandelia homologous gene using *K. candel* as an outgroup. Genes with *p*-value lower than 0.05 in the one side Fisher’s exact test with Benjamini-Hochberg multiple test (Benjamini & Hochberg, 1995) correction were classified as positively selected genes.

### 2.7 Genotype-environment associations

Redundancy analysis (RDA) was used to identify associations between SNP variations across diverse populations and environmental parameters (Forester et al., 2018). Climate data including the mean annual temperature, mean annual minimum temperature, and mean annual precipitation for the three sampling locations were obtained from the Central Meteorological Observatory (http://www.nmc.cn/). The RDA was conducted using the RDA function from the vegan package (Oksanen et al., 2007) with the input file of genotype matrix comprising all SNPs as transformed by PLINK v1.90. Genotypes at each SNP site was encoded as follows: 0 for homozygotes identical to the reference, 1 for heterozygotes, and 2 for homozygotes differing from the reference. The proportion of variance explained by the environmental variables was evaluated using the RsquareAdj function. The significance of the linear relationship between each constrained axis and the environmental variables was then assessed using the anova.cca function. In this study, two constrained axes (RDA1 and RDA2) were found to be significant. Outlier SNPs for each significant constrained axis were identified using a cutoff of 1.96 SD greater or less than mean (two-tailed *p*-value = 0.05) and were annotated with known genes based on the *K. obovata* genome.

### 2.8 *K. obovata* gene functional annotation and GO enrichment analysis

To annotate the *K. obovata* genes, BlastP (Altschul et al., 1990) was used to identify homologous genes between the *K. obovata* and *Arabidopsis thaliana* using an *e*-value threshold of < 1e-10. Gene Ontology (GO) functions of each *K. obovata* gene were annotated using eggNOG-mapper (Cantalapiedra et al., 2021). To evaluate potential over-representation of functional gene classes, we conducted GO enrichment analysis using the R package ClusterProfiler (Yu et al., 2012) by applying the annotation mentioned above. The statistical significance of over-represented GO terms within the input gene sets was assessed through Fisher’s exact tests, with a significance threshold set at False Discovery Rate (FDR) < 0.2.

## 3 Results

### 3.1 Genomic data and genetic diversity

**Figure 1:**
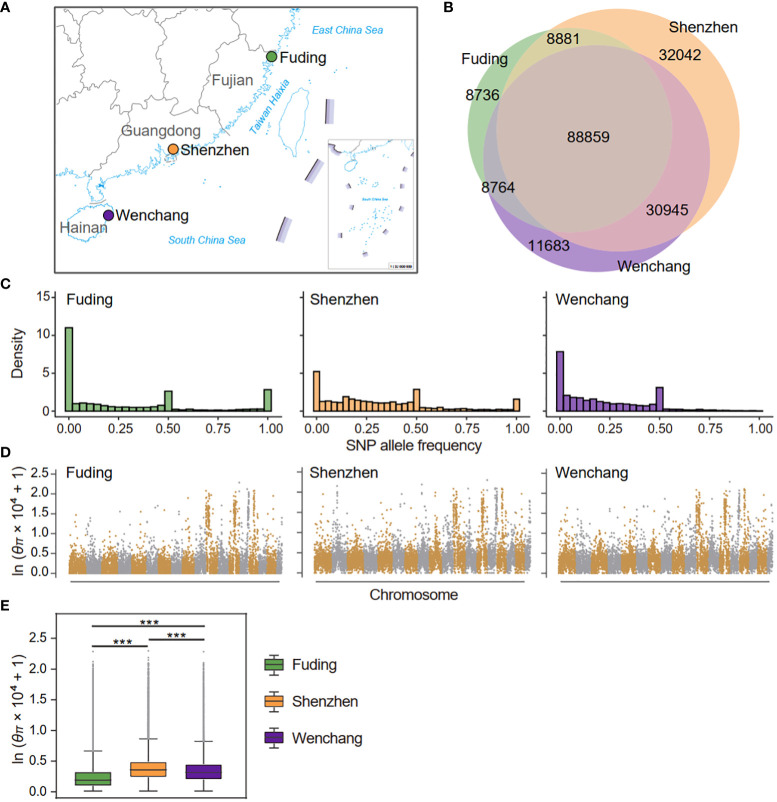
Sampling locations and genetic diversity of three populations of *Kandelia obovata* in China. **(A)** Map depicting the sampling locations of the Fuding (*n* = 14), Shenzhen (*n* = 17), and Wenchang (*n* = 15) populations in China. **(B)** Venn diagrams show the numbers of shared and private SNPs detected in the three *K obovata* populations. **(C)** Site frequency spectra based on 189,909 SNPs in each population. **(D)** Genome-wide analysis of nucleotide diversity (*θ_π_*) in each *K obovata* populations. *θ_π_* was calculated in nonoverlapping 20-kb bins and displayed in logarithmic scale across the *K obovata* genome. **(E)** Boxplot displaying the distribution of *θ_π_* in three *K obovata* populations. Asterisks indicate the significance level of Mann-Whitney U test: ***p-value < 0.001.

A total of 0.82 Tb of data with an average sequencing depth of approximately 40× per individual were obtained for the 46 K*. obovata* individuals from the three populations, representing the natural distribution of *K. obovata* from north to south in China (**Table 1**; **Figure 1A**). After quality control and filtering, the clean reads were mapped to the reference genome of *K. obovata*, achieving an average mapping rate of 93.33%. The average depth of uniquely mapped reads per site was 40.23 (**Supplementary Table S1**). In total, we obtained 189,909 high-confidence single nucleotide polymorphism (SNP) sites, corresponding to an average density of 1.07 SNPs per kilobase in the *K. obovata* genome. Out of these SNPs, 25,988 (13.7%) were in protein-coding regions, including exons and introns, 27,551 (14.5%) were found in putative regulatory regions, including promoters (2kb upstream of TSS) and UTRs, and the majority (56.7%) were intergenic (**Supplementary Figure 2**).

**Table 1:** Sampling information and genetic measures of three *K. obovata* populations.

Terms	Populations		
	Fuding (F)	Shenzhen (S)	Wenchang (W)
Location (longitude, latitude)	120.33°E, 27.29°N	114.52°E, 22.65°N	110.83°E, 19.60°N
Population size	14	17	15
Annual average temperature (°C)	19.5	23.5	24.9
Annual average minimum temperature (°C)	5.8	12.4	15.6
Annual average precipitation (mm)	1,743.3	1,889.3	1,913.0
Number of SNPs	115,239	160,726	140,250
Nucleotide diversity (*θ_π_*)	2.32 × 10^-4^	3.27 × 10^-4^	2.87 × 10^-4^

When comparing between populations, the number of high-confidence SNP sites was highest for the Shenzhen population (160,726), followed by the Wenchang (140,250) and Fuding (115,239) population. The number of private or population-specific SNPs were 8,736, 32,042, and 11,683 for the Fuding, Shenzhen and Wenchang populations, respectively (**Figure 1B**). The site frequency spectrum of individual *K. obovata* populations revealed that the Fuding populations had the highest proportion (16.7%) of fixed SNPs followed by the Shenzhen population (5.5%), while only 75 SNPs (0.6%) were fixed in the Wenchang population (**Figure 1C**). However, the Wenchang population had a higher percentage (9.4%) of rare SNPs (frequency < 0.05), than the Fuding (5.8%) and Shenzhen population (4.4%, **Figure 1C**). Meanwhile, all three populations possessed considerable medium-frequency SNPs, indicating a high level of heterozygosity within individuals of *K. obovata* (**Figure 1C**).

We divided the *K. obovata* genome into 20-kb non-overlapping bins and used Tajima’s π (*θ_π_*) as a measurement of nucleotide diversity at the individual SNP level. The average nucleotide diversity per site was calculated for each bin and compared between populations of *K. obovata*. Nucleotide diversity was unevenly distributed across the *K. obovata* genome, with the most pronounced diversity observed in chromosomes 11, 13, 14 and 15 (**Figure 1D**). Consistent with the observed number of high-quality SNPs, the Shenzhen population exhibited the highest nucleotide diversity averaged across the genome (mean ± SD, *θ_π_* = 3.27 × 10^-4^ ± 9.63 × 10^-4^), followed by the Wenchang population (*θ_π_* = 2.87 × 10^-4^ ± 8.64 × 10^-4^), while the Fuding population showed the lowest nucleotide diversity (*θ_π_* = 2.32 × 10^-4^ ± 8.87 × 10^-4^) (**Figure 1E**). The differences in nucleotide diversity between pairwise populations were all significant (Mann-Whitney U test, all *p*-value < 0.05; **Figure 1E**).

### 3.2 Genetic differentiation and population structure

**Figure 2:**
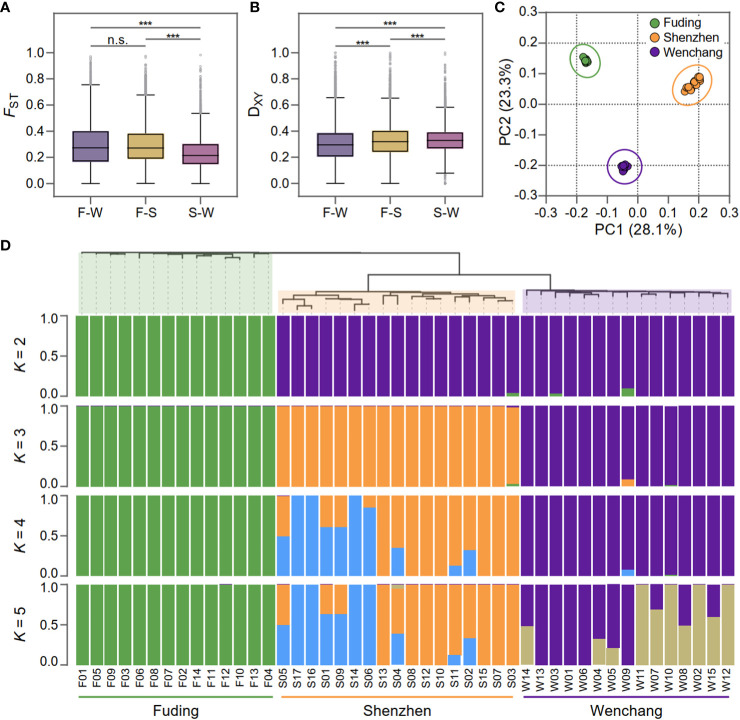
Population differentiation and genetic structure of *Kandelia obovata*. **(A) ** Boxplot displaying the genome-wide distributions of fixed index (*F*_ST_) in three pairwise populations: F-W (Fuding vs. Wenchang population), F-S (Wenchang vs. Shenzhen population), and S-W, (Wenchang vs. Shenzheng population). **(B) ** Boxplot displaying genome-wide distributions of genetic divergence (D_XY_) in the same three pairwise populations. Asterisks indicate the significance level of Mann-Whitney U test: ***, *p*-value < 0.001; “n.s.”, non-significant. **(C) ** Principal components analysis (PCA) based on 189,909 SNPs showing genetic separation among the 46 *K. obovata* samples. Principal components 1 (28.1%) and principal components 2 (23.3%) are shown. **(D)** Phylogenetic tree of individuals and population genetic structure. Each individual is represented by a vertical bar, which is partitioned into *K* (*K* = 2, 3, 4, and 5) colored segments reflecting the individual's probability of membership to each genetic cluster.

To assessed population differentiation, the fixation index *F*_ST_ (Weir and Cockerham, 1984) and the average nucleotide diversity D_XY_ (Nei & Miller, 1990) were calculated for each SNP and averaged within each bin for pairwise populations of *K. obovata* (**Figure 2**). *F*_ST_ values revealed substantial population differentiation, particularly evident when comparing the Fuding population with the others: Fuding vs. Shenzhen (*F*_ST_ = 0.30 ± 0.19) and Fuding vs. Wenchang (*F*_ST_ = 0.30 ± 0.16), in contrast to Shenzhen vs. Wenchang (*F*_ST_ = 0.24 ± 0.13) (**Table 1** and **Figure 2A**). D_XY_ values were similar among pairs of populations (D_XY_ = 0.30 ± 0.15 to 0.33 ± 0.11), all supporting a high level of population differentiation in *K. obovata* (**Figure 2B**). The principal component analysis (PCA) revealed a clear clustering pattern, indicating that individuals within a population tend to group together and are distinctly separated from individuals in other populations (**Figure 2C**). Similarly, the structure analysis indicated that the pattern of ancestry was best represented by *K* = 3, which is supported by the lowest minimum *K*-value error rate (cross-validation error = 0.41) (**Figure 2D**). The three clusters that resulted from *K* = 3 clearly separated individuals according to their population origination. When *K* = 2, individuals from Fuding population formed a cohesive cluster, while individuals from Shenzhen and Wenchang clustered together. Subsequent analyses at *K* values of 4 and 5 revealed highly variable levels of admixture within the Shenzhen and Wenchang populations. However, there is no significant admixture within the genetic cluster in Fuding population, indicating a relatively more isolated genetic profile for this population.

### 3.3 Inference of demographic history

**Figure 3:**
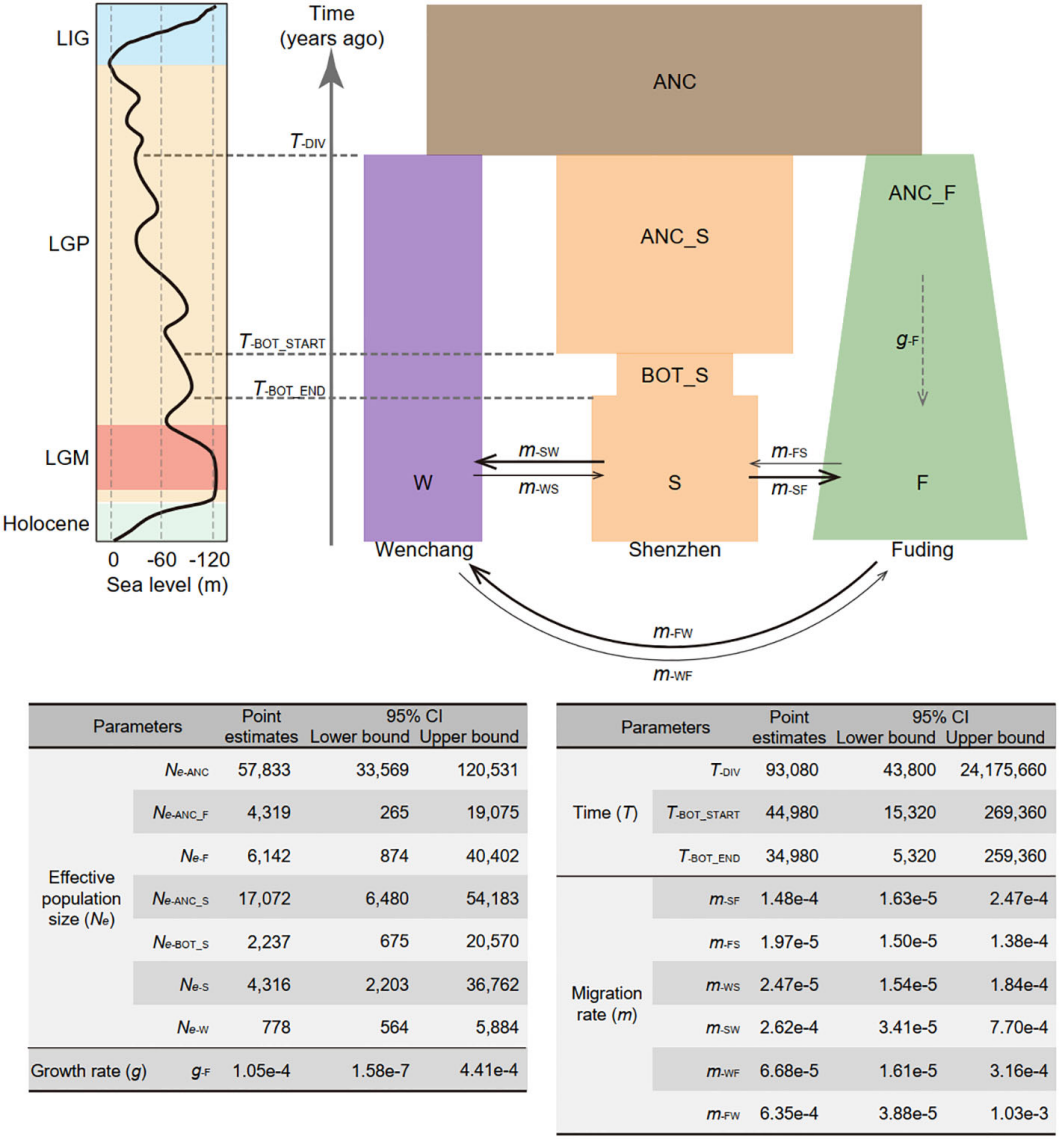
Demographic model depicting the population history of *Kandelia obovata* in China. Populations are represented by rectangles in distinct colors: ancestral population (ANC) in brown, Wenchang population (W) in purple, Shenzhen population (S) in orange, and Fuding population (F) in green. Changes in the width of each rectangle reflects changes in population size. Solid arrows denote gene flow between pairwise populations, with arrow direction indicating the direction of gene flow. The dashed arrow signifies population expansion in the Fuding population. Point estimates of demographic parameters, including effective population size (*N_e_*), population size growth rate (*g*), time (*T*), and migration rate (*m*), along with their 95% confidence intervals (CI), are presented below the demographic model. The parameters were estimated using a neutral mutation rate per site per generation (*µ*) of 7.86 × 10^−8^ and a generation time of 20 years for *K. obovata*. The line chart on the left illustrates the relative sea levels during the last glacial period, depicting the last interglacial period (LIG), the last glacial period (LGP), and the last glacial maximum (LGM).

The optimal demographic model (model 15; log-likelihood = -1089415.63, AIC = 2178865.27, ΔAIC = 0), as shown in **Figure 3** and **Supplementary Table 3**, supports a composite history inclusive of divergence, bottleneck, expansion, and migration of *K. obovata.* With a generation time of 20 years and mutation rate of 7.86 × 10^−8^ per site per generation (He et al., 2022), the optimal demographic model suggests that the three *K. obovata* populations diverged from their ancestral population with an effective population size (*N_e_*) of 57,833 (95% CI: [33,569, 120,531]) approximately 93,080 years ago (95% CI: [43,800, 24,175,600]). This coincides the late onset of the last glacial period, marked by highly unstable sea levels. Subsequently, the Fuding population underwent a gradual population expansion with a growth rate of 1.05e-4 (95% CI: [1.58e-7, 4.41e-4]), increasing from an *N_e_* of 4,319 (95% CI: [265, 19,075]) to 6,142 (95% CI: [874, 40,402]). In contrast, the ancestral Shenzhen population, with an *N_e_* of 17,072 (95% CI: [265, 19,075]), experienced a ten-thousand-year bottleneck around 45,000 years ago (95% CI: [15,320, 269,360]), coinciding with the start of the last glacial maximum. The Wenchang population maintained a consistently small value of *N_e_* of 778 (95% CI: [564, 5,884]) over the last hundred thousand years with minimal fluctuation. Migration rates per generation between populations varied substantially, with gene flow mainly occurring from Fuding to Wenchang (*m*_FW_) at a rate of 6.35e-4 (95% CI: [3.88e-5, 1.03e-3] and from Shenzhen to Wenchang (*m*_SW_ = 2.62e-4, 95% CI: [3.41e-5, 7.70e-4]) or Fuding (*m*_SF_ = 1.48e-4, 95% CI: [1.63e-5, 2.47e-4]), while the others were relatively small (**Figure 3**).

Notably, both the Shenzhen and Fuding populations have experienced changes in population size according to the best model. Despite high genetic diversity, a model that assumed a bottleneck occurred in the Shenzhen population (model 6; log-likelihood = -1091480.43, AIC = 2182978.86, ΔAIC = 4113.594) was more likely than one assuming a bottleneck in the Fuding population (model 7; log-likelihood = -1091661.914, AIC = 2183341.828, ΔAIC = 4476.562) (**Supplementary Figure 1** and **Supplementary Table 3**). In contrast, although the Fuding population is marginal, a model that assumed expansion in the Fuding population (model 10; log-likelihood = -1091358.265, AIC = 2182730.53, ΔAIC = 3865.26) was more likely than in the other two populations (**Supplementary Figure 1** and **Supplementary Table 3**).

### 3.4 Detection of positive selection

**Figure 4:**
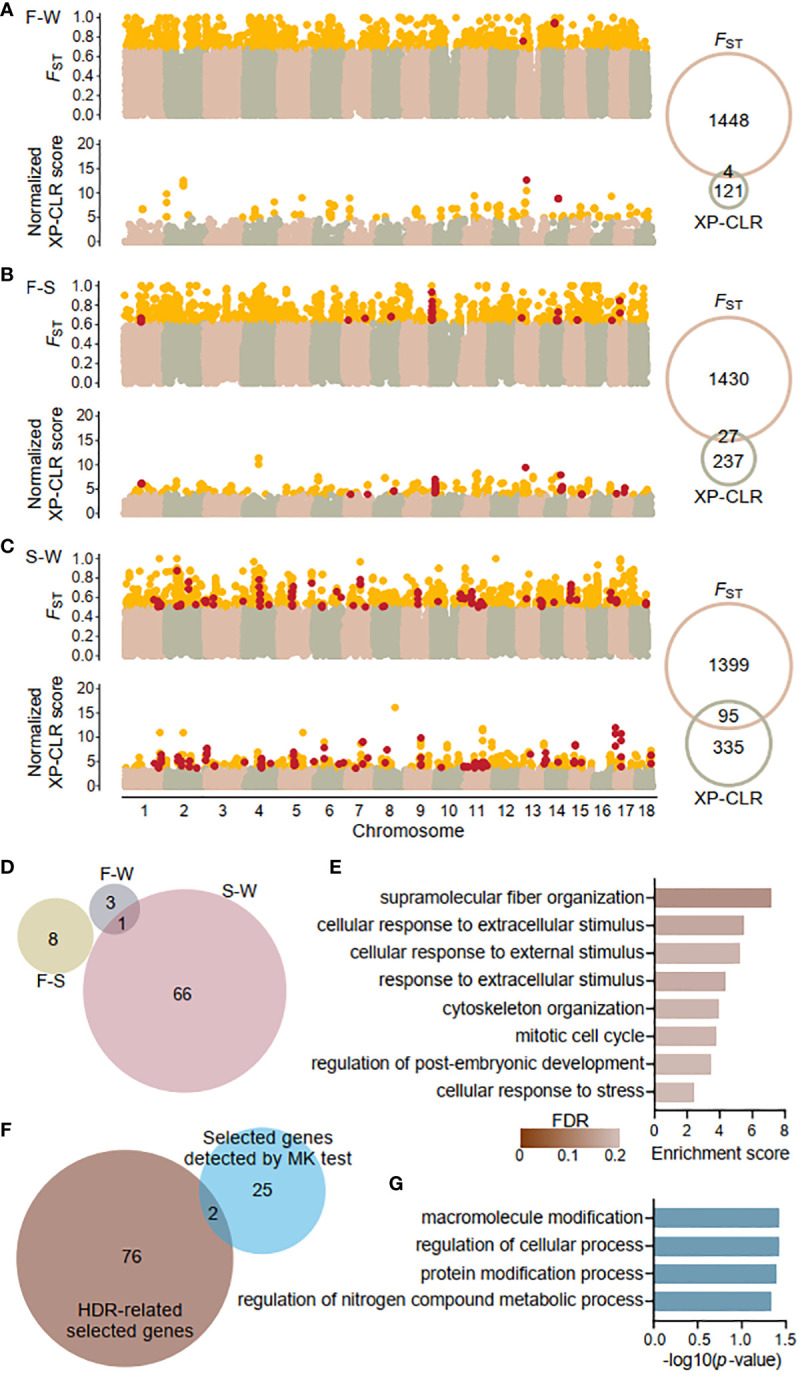
Genome wide signatures of selection and selected genes. Sliding window analysis of fixation index (*F*_ST_) and cross-population composite likelihood ratio (XP-CLR) with 20-kb window size and 5-kp step size across the *K obovata* genome for pairwise populations: **(A)** Fuding and Wenchang population (F-W), **(B)** Fuding and Shenzhen population (F-S), and **(C)** Shenzhen and Wenchang population (S-W). Outlier values (defined as at least 1.96 SD above the mean) are indicated in gold. Venn diagrams show the number of outliers identified by each method. Windows exhibiting both *F*_ST_ and XP-CLR outliers were identified as highly differentiated regions (HDRs) and indicated in red. **(D)** Venn diagrams showing the numbers of genes residing in HDRs that were identified in the three comparison pairs. All 78 genes identified as HDR-related selected genes are listed in **Supplementary Table S4**. **(E)** Bar plot displaying the enriched Gene Ontology (GO) terms of biological process of HDR-related selected genes. **(F)** Venn diagrams showing the overlaps between the 78 HDR-related selected genes and the 27 selected genes identified by the McDonald-Kreitman test (MK test). Genes detected by MK test are listed in **Supplementary Table S5**. **(G)** Bar plot displaying the enriched GO terms of biological process of 27 selected genes identified by the MK test.

Sliding widow analysis of population differentiation used a cutoff of at least 1.96 SD above the mean (*p* < 0.025) to detect highly differentiated outliers for both *F*_ST_ and XP-CLR values. For the *F*_ST_ values, 1452, 1457, and 1494 outliers were detected for three pairwise comparisons: Fuding and Wenchang (F-W), Fuding and Shenzhen (F-S), and Shenzhen and Wenchang (S-W), respectively (**Figures 4A–C**). For XP-CLR values, 125, 264, and 430 outliers were detected for the same pairwise comparisons (**Figures 4A–C**). The regions where both methods detected outliers were considered as the highly differentiated regions (HDRs) or the candidate regions for selection. Sliding window analysis of Fay and Wu’s H-statistic (Fay & Wu, 2000), designed to detect high-frequency hitchhiking alleles associated with selective sweeps, revealed significantly lower *H* values in HDRs than in the whole genome for each of the three populations (Mann-Witney U test, all *p*-value < 0.001; **Supplementary Figure 3**), suggesting selective sweeps occurred in HDRs of each population. Negative *H* values were observed in HDRs of the Fuding (mean ± SD, *H*-statisic= -2.43 ± 3.22) and Shenzhen populations (*H*-statistic = -2.43 ± 2.12), but not in the Wenchang population (*H*-statistic= 0.13 ± 1.03; **Supplementary Figure 3**), indicating that selective signatures of HDRs are stronger in the two northern populations compared to the Wenchang population.

A total of 4, 27, and 95 HDRs were identified for the F-W, F-S, and S-W comparisons, respectively, comprising 4, 8, and 67 genes and a total of 2,868 SNPs (**Figure 4D**; **Supplementary Table 4**). Among them, 77 genes were identified in a single population pair, while one gene, *Plant U-box 45* (*PUB45*; Maker00002595), was shared by the F-W and S-W comparisons. Notably, no genes were shared by all three comparisons, resulting in a total of 78 genes in HDRs (**Figure 4D**). Furthermore, Gene Ontology (GO) analysis revealed that the identified genes are enriched in functional categories of supramolecular fiber organization (GO: 0097435) and those related to response to stimuli, including cellular response to extracellular stimulus (GO: 0031668), cellular response to external stimulus (GO: 0071496), response to extracellular stimulus (GO: 0009991), and cellular response to stress (GO: 0033554). Additionally, other enriched functional categories are involved in cell cycles and the regulation of developmental process (**Figure 4E**).

Using *K. candel* as an outgroup, we also detected genes under positive selection by the McDonald-Kreitman (MK) test (McDonald & Kreitman, 1991). Applying the G test of independence described in the original MK test, we identified 27 genes with a positive selection signature at a cutoff of an adjusted *p*-value < 0.05 (**Supplementary Table S5**). Only two genes, *AP2-like ethylene-responsive transcription factor 1* (*AIL1*; Maker00004062) participating in the ethylene-activated signaling pathway (Kim et al., 2006) and *callose synthase 5* (*CALS5*; Maker00007136) involved in the regulation of pollen tube growth (Dong et al., 2005) were also found in the list of HDRs-related selected genes (**Figure 4F**). The 27 candidate genes were enriched in housekeeping functional categories including macromolecule modification (GO: 0043412), regulation of cellular process (GO: 0050794), protein modification process (GO: 0036211), and regulation of nitrogen compound metabolic process (GO: 0034641) with a significance threshold of *p*-value < 0.05 (**Figure 4G**). However, none of these overrepresentations retained significant after FDR correction, likely due to the limited size of gene list.

### 3.5 Genotype-environment associations

**Figure 5:**
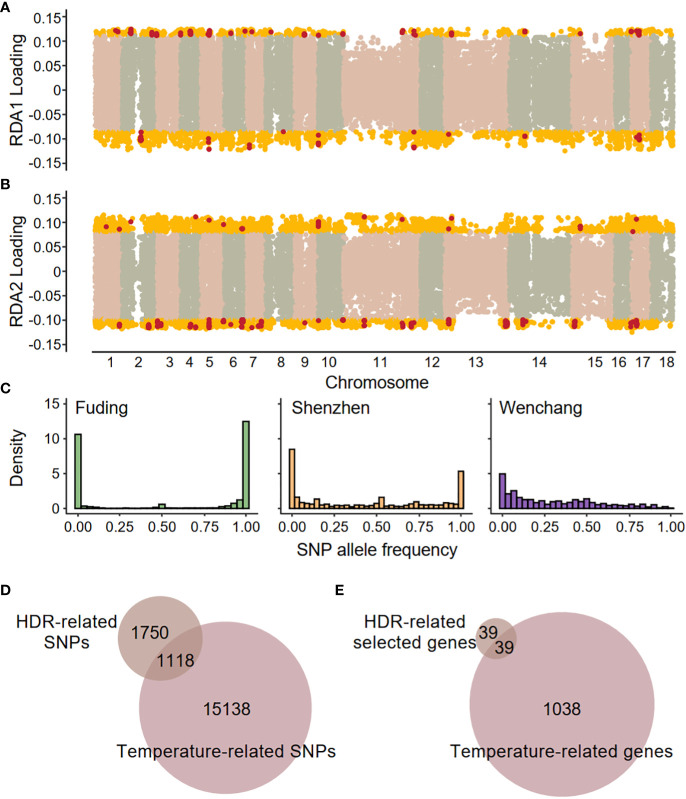
Genome–environment associations detected by redundancy analysis (RDA). SNP loadings on **(A)** the first RDA axis (RDA1) and **(B)** the second RDA axis (RDA2). The gold dots represent SNPs with significant associations along the RDA axes (defined as 1.96 standard deviations below or above the mean), and these SNPs are identified as temperature-related SNPs. The red dots represent SNPs located in highly differentiated regions (HDRs). **(C)** Site frequency spectra based on 16,256 temperature-related SNPs in each population. **(D)** Venn diagrams showing the overlaps between HDR-related SNPs and temperature-related SNPs. **(E)** Venn diagrams showing the overlaps between HDR-related selected genes and temperature-related genes. A total of 39 shared genes were identified as high-confidence candidate genes responsible for local temperature adaptation and are listed in **Table 2**.

The three populations, located at different latitudes, differ in their local environments. Redundancy analysis (RDA) detected significant associations between SNPs and two environmental variables (F-statistics, both *p*-value < 0.001), the mean annual temperature (MAT) and the mean annual minimum temperature (MAMT), but not between SNPs and the mean annual precipitation (MAP, **Supplementary Figure 4A**). This suggests that temperature is the primary environmental variable shaping population differentiation. Two constrained axes (RDA1 and RDA2) explained about 29% of the total variation. Using a cutoff of 1.96 SD greater or less than mean (two-tailed *p*-value = 0.05), a total of 16,256 environment-associated SNPs were identified as candidate SNPs involved in local adaptation (**Figure 5**; **Supplementary Figure 4B**). The temperature-related SNPs exhibited distinct patterns of allele frequency spectrum compared to genome-wide SNPs in all three populations (Kolmogorov-Smirnov test, *p*-value < 2.2 × 10^-16^ for all comparisons), characterized by an elevated proportion of fixed SNPs in the Fuding and Shenzhen populations (**Figure 5C**). Among the 16,256 temperature-associated SNPs, 1,118 were located in the HDRs, indicating a significant enrichment of temperature-associated SNPs in HDRs compared to the whole genome (χ^2^ test, *p*-value < 2.2 × 10^-16^; **Figure 5D**). These temperature-related SNPs are within 1,074 genes which were enriched in a range of functional categories involved in sexual reproduction, DNA repair, cell death, cell cycle and immune response (**Supplementary Table S6**).

A total of 39 genes containing temperature-associated SNPs within their exons and located within HDRs were finally identified (**Figure 5E**). These genes, consisting of 2, 6, and 32 for the F-W, F-S, and S-W comparisons, respectively, were considered as high-confidence candidates underlying local adaptation to temperature in *K. obovata* (**Table 2**). In the F-W comparison, two genes were identified, namely *Plant U-box 45* (*PUB45*; Maker00002595) and a gene (Maker00006413) containing eight temperature-associated SNPs but no homolog in *Arabidopsis* (**Table 2**). *Plant U-box 45*, known for its function in protein ubiquitination and cold response (He et al., 2023), was also identified as a high-confidence candidate in the S-W comparison. Notably, the high-confidence candidates in the F-S comparison with known functions included *CRY2 interacting splicing factor 1* (*CIS1*; Maker00002948) involved in the regulation of flowering (Zhao et al., 2022), *tRNA pseudouridine synthase A 5* (*TRUA5*, Maker00014866) involved in RNA modification, *Pol-like 5* (*PLL5*; Maker00017856) participating in leaf development (Song & Clark, 2005), a 2-oxoglutarate (2OG) and Fe(II)-dependent oxygenase superfamily protein (Maker00003467), and a UDP-N-acetylglucosamine (UAA) transporter family gene (Maker00018193). Meanwhile, for the S-W comparison, candidate genes were found to be responsive to cold and other stresses, such as *growth-regulating factor 5* (*GRF5*; Maker00004032; Lantzouni et al., 2020), *ATP-binding cassette G40* (*ABCG40*; Maker00008456; Baron et al., 2012) and *Integrin-linked kinase 1* (*ILK1*; Maker00007966; Brauer et al., 2016), or participate in various development processes, such as *cryptochrome 1* (*CRY1*; Maker00010750) involved in flowering (Gao et al., 2023), *cotton Golgi-related 2* (*CGR2*; Maker00005232) involved in leaf morphogenesis (Kim et al., 2015) and *lonesome highway* (*LHW*; Maker00018105) involved in root development (Ohashi-Ito & Bergmann, 2007) (**Table 2**).

**Table 2:** High-confidence candidate genes underlying local adaptation in *K. obovata*.

*K. obovata* gene	Populations	Number of temperature-related SNPs	Orthologs in *Arabidopsis*	Gene symbol	Description	Main GO terms of biological process
Maker00002595	F-W, S-W	2	AT1G27910	*PUB45*	Plant U-box 45	protein ubiquitination
Maker00006413	F-W	8			Carbonic anhydrase 2	carbon utilization
Maker00002948	F-S	1	AT3G52120	*CIS1*	CRY2 interacting splicing factor 1	RNA processing
Maker00003467	F-S	1	AT5G48020		2-oxoglutarate (2OG) and Fe(II)-dependent oxygenase superfamily protein	
Maker00014866	F-S	1	AT5G35400	*TRUA5*	Enzyme for the pseudouridine (Ψ) to uridine (U) conversion	tRNA pseudouridine synthesis
Maker00017856	F-S	1	AT1G07630	*PLL5*	Pol-like 5, protein phosphatase 2C like gene	protein dephosphorylation
Maker00017915	F-S	1				
Maker00018193	F-S	16	AT5G59740		UDP-N-acetylglucosamine (UAA) transporter family	transmembrane transport
Maker00000528	S-W	2	AT3G02750		Protein phosphatase 2C family protein	peptidyl-threonine dephosphorylation
Maker00000994	S-W	1			Iron ascorbate-dependent oxidoreductase family	
Maker00001853	S-W	1	AT4G14300	*RBGD4*	RNA-binding glycine-rich protein D4	regulation of response to salt stress
Maker00004032	S-W	1	AT3G13960	*GRF5*	Growth-regulating factor 5	response to cold
Maker00004062	S-W	6	AT1G72570	*AIL1*	AP2-like ethylene-responsive transcription factor	ethylene-activated signaling pathway
Maker00004523	S-W	1	AT3G25905	*CLE27*	CLAVATA3/ESR-Related 27	cell-cell signaling involved in cell fate commitment
Maker00004536	S-W	1				
Maker00004832	S-W	8	AT4G02210		Myb/SANT-like DNA-binding domain protein	
Maker00005232	S-W	10	AT3G49720	*CGR2*	Cotton Golgi-related 2	leaf morphogenesis
Maker00005452	S-W	1	AT1G50060		CAP superfamily protein	biological process
Maker00005494	S-W	1	AT4G33630	*EX1*	Executer 1	response to singlet oxygen
Maker00006074	S-W	1	AT5G64380		Inositol monophosphatase family protein	fructose metabolic process
Maker00006210	S-W	1	AT1G08980	*AMI1*	Amidase 1	auxin biosynthetic process
Maker00006424	S-W	1	AT2G03480	*QUL2*	Quasimodo2 like 2	
Maker00006950	S-W	15	AT3G27350		Transcriptional regulator ATRX-like protein	
Maker00007136	S-W	3	AT2G13680	*CALS5*	Callose synthase 5	regulation of pollen tube growth
Maker00007772	S-W	1	AT1G65540	*LETM2*	Leucine zipper-EF-hand-containing transmembrane protein 2	
Maker00007966	S-W	11	AT2G43850	*ILK1*	Integrin-linked kinase 1	response to osmotic stress
Maker00008456	S-W	16	AT1G15520	*ABCG40*	ATP-binding cassette G40	response to abscisic acid
Maker00010492	S-W	1	AT2G44970		Alpha/beta-Hydrolases superfamily protein	
Maker00010750	S-W	10	AT4G08920	*CRY1*	Cryptochrome 1	response to blue light
Maker00012336	S-W	1	AT2G35630	*MOR1*	Microtubule organization 1	cell plate formation involved in plant-type cell wall biogenesis
Maker00013529	S-W	1	AT1G04140		Transducin family protein/WD-40 repeat family protein	regulation of protein catabolic process
Maker00015311	S-W	1	AT5G42570		B-cell receptor-associated 31-like protein	intracellular protein transport
Maker00015660	S-W	1	AT1G05100	*MAPKKK18*	Mitogen-activated protein kinase kinase kinase 18	negative regulation of stomatal opening
Maker00016003	S-W	2	AT5G45360	*SKIP31*	SKP1-interacting partner 31	protein ubiquitination
Maker00016879	S-W	1	AT4G33925	*SSN2*	Suppressor of sni1 2	defense response
Maker00018105	S-W	1	AT2G27230	*LHW*	Lonesome highway	root development
Maker00018163	S-W	5	AT5G22100		RNA cyclase family protein	ribosome biogenesis
Maker00018375	S-W	1	AT4G23740		Leucine-rich repeat protein kinase family protein	
Maker00018456	S-W	1	AT4G23730		Galactose mutarotase-like superfamily protein	carbohydrate metabolic process

## 4 Discussion

*K. obovata* offers valuable opportunities for investigating the genetic basis of local adaptation in species that have undergone ecological speciation with complex demographic histories. Here, by examining how genome-wide patterns of population structure relate to genotype-environment associations, in conjunction with inferring demographic history and detecting selection signatures, we empirically contribute to the broader understanding of how genetic variation leads to climate adaptation. Despite complex demographic history, our results demonstrate that natural selection drives local adaptation in *K. obovata* due to differential selective pressures related to temperature.

### 4.1 Complex demography and restricted gene flow

The complex demographic history of the *K. obovata* populations has a significant impact on their genetic diversity and population structure. The Fuding population, occurring in the northmost in China, exhibits the lowest genetic diversity and is more isolated from the other two populations. This is consistent with the expectation for marginal populations, which often show lower genetic diversity and increased genetic differentiation from more central populations (Eckert et al., 2008). However, the inference of the demographic model revealed that the Fuding population has experienced a mild population expansion, while the central population in Shenzhen underwent a population bottleneck around the start of the last glacial maximum (**Figure 3**). These divergent patterns of population size change may align with the cold-tolerant nature of *K. obovata*, with the northern population being better adapted to cold weather than the southern population (Lu et al., 2022). Additionally, these two populations exhibit distinct patterns in the site frequency spectrum compared to the Wenchang population, with the former two showing a high number of fixed SNPs while the Wenchang population has almost none (**Figure 1C**). These results suggest that genetic drift associated with frequent population size change in the Fuding and Shenzhen populations may have caused rapid fixation of alleles in these regions, while the southern population in Wenchang may represent the ancestor state of this species, considering that *K. obovata* is diverged from the cold intolerant *Kandelia candel* (Sheue et al., 2003). Nevertheless, it is unclear how the Wenchang population could maintain a constant population size, especially given that global cooling during the last glacial maximum is known to be a major cause of extinction for tropical woody species such as mangroves (Song et al., 2021).

It is widely acknowledged that mangroves exhibit long-distance gene flow due to the dispersal of floating propagules (Van der Stocken et al., 2022). Previous studies on *K. candel* have indicated substantial regional gene flow over considerable distances (Chiang et al., 2001; Geng et al., 2008). In this study, the estimated gene flow from Fuding to Wenchang (*Nm*_FW_ = 3.90) is much greater than that from Wenchang to Fuding (*Nm*_WF_ = 0.05), despite the broad confidence intervals (**Figure 3**). This finding is consistent with the direction of current flow in the South China Sea during early winter (Shaw & Chao, 1994), coinciding with the maturation and shedding of the hypocotyl in *K. obovata* in the northern regions (Khoon et al., 2004). Thus, the asymmetric migration between the Fuding and Wenchang populations provides additional evidence for the influence of ocean currents on gene flow in mangroves. However, we observed strong population structure among the three *K. obovata* populations in China (**Figure 2D**), indicating local gene flow may be insufficient to counteract genetic differentiation caused by either genetic drift or natural selection. Notably, the best-fitting demographic model (**Figure 3**) reveals substantial gene flow occurring only from the Fuding and Shenzhen populations to the Wenchang population (*Nm*_FW_ = 3.90 and *Nm*_SW_ = 1.13), while all other *Nm*estimates, ranging from 0.02 to 0.64, are relatively small. Contrastingly, a recent study on grey mangrove (*Avicennia marina*) populations across the Red Sea, the Arabian Sea and the Persian/Arabian Gulf revealed a moderate population genetic structure correlating with geographic distance, which supports clades both among and within the seas surrounding the Arabian Peninsula (Friis et al., 2024).

What might have caused the limited gene flow among geographically proximate *K. obovata* populations? One possible explanation is the impact of human activities, which have led to increased fragmentation. Human activities such as land development, urbanization, and habitat destruction can create barriers to gene flow, resulting in population isolation and reduced genetic exchange between populations (Guo et al., 2016). Moreover, the small effective population size for all populations in this study may have also played a role in limiting gene flow. With a small effective population size, genetic drift has a more pronounced effect, leading to increased genetic differentiation between populations (Song et al., 2006). This effect may be particularly significant for the Shenzhen and Fuding populations, which have experienced population bottleneck and expansion, respectively.

### 4.2 Local adaptation and genotype-environment associations

Local adaptation involves significant changes in allele frequency. Therefore, conducting a genome scan to identify outlier values of the allelic differentiation is the preferred method for detecting loci associated with local adaptation. It has been recognized that high neutral differentiation among populations can make it more difficult to detect high outlier loci (Pérez-Figueroa et al., 2010). This is evident in the case for *K. obovata*, where the average *F*_ST_ values are approximately 0.3 across the genome (**Figure 2B**). Utilizing the 1.96 SD cutoff of the empirical distribution, we discovered that the number of *F*_ST_ outliers was largely comparable for all three pairwise population comparisons, whereas the number of XP-CLR outliers varied substantially among comparisons (**Figure 5A-C**). Given that *F*_ST_ is the quotient of two variances, the large expected variability in *F*_ST_ among neutral loci, influenced by complex demography, population structure, and migration, may impact the power to discern high outlier *F*_ST_ values, potentially resulting in a high rate of false positive or false negative (Le Corre & Kremer, 2012). In contrast, the XP-CLR method, which is independent of window size and robust to uncertainty regarding demography history, identifies regions in the genome where the change in allele frequency at the locus occurred too quickly to be due to random drift (Chen et al., 2010). The different results between the two methods lies in the fact that the detection of *F*_ST_ outliers depends on neutral loci, while the XP-CLR method is sensitive to recent selective sweeps. By combining both methods, we observed an enrichment of environment-associated SNPs in highly differentiated regions (HDRs) that showed both outlier *F*_ST_ and outlier XP-CLR values, indicating the effectiveness of our analyses in identifying loci associated with local adaptation in the presence of high neutral differentiation among populations.

The three *K. obovata* populations, located at varying latitudes, are subject to differing intensities of selective pressure. Genotype-environment associations in *K. obovata* were detected exclusively for temperature-related variables, indicating that temperature is the primary factor driving differential selective pressures leading to local adaption. This supports the notion that winter temperature shapes mangrove distributions and assemblage composition in China (Wu et al., 2018). Local temperature has been consistently identified as significant drivers of varying selective pressures in many organisms, such as grey mangrove (Friis et al., 2024), lichen-forming fungi (Valim et al., 2023), and mountain pine (Méndez-Cea et al., 2023). In this study, the Fuding and Shenzhen populations showed a higher proportion of fixation in temperature-associated SNPs (**Figure 5C**) compared with genome-wide SNPs (**Figure 1C**), implying that natural selection has facilitated the rapid evolution of genes related to local adaptation. However, the evidence for local adaptation in the Wenchang population is weak, as reflected by the allele frequency spectra (**Figure 5C**) and results of Fay and Wu’s *H* (**Supplementary Figure 3**), likely due to the minimal selective pressure for temperature adaptation in this tropical region.

Gene flow may have also impacted local adaptation in the Wenchang population, which receives migrates from the Fuding and Shenzhen populations (*Nm*_FW_ = 3.90 and *Nm*_SW_ = 1.13; **Figure 3**). Contrary to expectations based on local adaptation gradients, many more HDRs were found between the Shenzhen and Wenchang populations than between the Fuding and Wenchang populations (**Figure 4C**). This inconsistency could stem from more substantial gene flow from Fuding to Wenchang compared to from Shenzhen to Wenchang (**Figure 3**). Both theoretical and empirical studies have shown that gene flow can either promote or counteract local adaptation depending on the extent of standing variation and the strength of natural selection (Tigano & Friesen, 2016). The scant number of selected candidate genes identified between the Wenchang and Fuding populations suggests that the weak selection in the Wenchang population is insufficient to overcome the homogenizing effect of gene flow. Nevertheless, we still identified a candidate gene under selection, *PUB45*, in both the comparison between the Fuding and Wenchang populations and the comparison between the Shenzhen and Wenchang populations (**Figure 4D**). This gene, which belongs to ubiquitin ligases enzymes, was reported to play a role in the cold acclimation of *K. obovata* seedlings in a previous study using transcriptome analysis (He et al., 2023). Collectively, our findings highlight the complexities involved in identifying loci responsible for local adaptation, especially in the presence of complex demographic histories, population structure, and gene flow.

### 4.3 Similarity and discrepancy between genes involved in local adaptation and those responsible for inter-species divergence

The question of whether the same genes responsible for local adaptation within a species also contribute to differences between species is a fundamental topic in evolutionary biology. *K. obovata* and its cold-intolerant relative *K. candel* are separated by the South China Sea, exhibiting genetic discontinuity and differential adaptation (Sheue et al., 2003). Among the 78 candidate genes within HDRs in *K. obovata*, only two genes, *AIL1* and *CALS5* overlap with those identified being under selection by the McDonald-Kreitman (MK) test when comparing *K. obovata* to *K. candel* (**Figure 4F**). This overlap may be due to both intra- and interspecific variations being influenced by similar selective pressures in cold environments. *AIL1* has been identified as an ethylene response factor and a transcription factor responsive to cold in grape (Ma et al., 2022). *CALS5* plays a crucial role in preserving the proper formation of callose walls during pollen development and responding to biotic stress (Dong et al., 2005). Evidence supporting the contribution of the same genetic variation to adaptive traits within and between species has been found in various organisms, such as antifreeze proteins in fish, indicating that the genetic mechanisms involved may be complex due to the polygenic nature of adaptive traits and the influence of gene-environment interactions (Berthelot et al., 2019).

The lack of overlap for the majority of candidate genes under selection, as identified by between-population comparisons with *F*_ST_ and XP-CLR and by inter-species comparison with MK test, can be explained by several factors. Firstly, intra-specific selection within *K. obovata* may target different genetic variations than inter-specific selection between *K. obovata* and *K. candel*. Although temperature adaptation is considered the primary force driving ecological speciation in Kandelia, the two species also differ in shoot, leaf, floral, fruit and hypocotyl characters, in addition to physiological differentiation (Sheue et al., 2003). The 27 selected genes detected by MK test are enriched in functional categories related to several protein modifications (**Figure 4G**). This is consistent with the notion that inter-specific selection often reflects more fundamental differences in niche occupation and overall lifestyle that have accrued over longer periods of evolutionary time (Tarjuelo et al., 2017). Second, the time scale of selection differs within and between species. Genes that were important in the initial divergence of the cold-tolerant *K. obovata* and cold-intolerant *K. candle* might not be the same genes that are currently under selection within *K. obovata*, which could be adapting to more recent or localized environmental changes. In line with this idea, four out of the top five enriched functional categories of the HDR candidate genes are associated with response to stimulus (**Figure 4E**). The discrepancies in genes identified as under selection between different methodologies may be a common issue (Balick et al., 2022; Wang X. et al., 2022; Benjelloun et al., 2023).

One limitation of this study is that only three *K. obovata* populations in China were surveyed. Considering the strong population structure among the surveyed populations (**Figure 2D**), we might capture both adaptive and neutral changes when identifying candidates under selection as high differential outliers between populations. Nevertheless, the enrichment of temperature-associated SNPs within HDRs indicates that most candidates are likely adaptive. On the other hand, the small number of populations surveyed may lead to an underestimation of genes responsible for local adaptation, as different environmental pressures might act on different gene interaction networks via gene-environment interactions (Tiffin & Ross-Ibarra, 2014). However, as temperature is the major factor shaping the evolutionary trajectories of Kandelia species, our results should capture the majority genetic variation critical for climate adaptation within and between species. A wider geographic sampling across the complete range of *K. obovata* and *K. candel* will be necessary to conduct further research on candidate functional loci associated with their ecological divergence.

## 5 Conclusion

Our investigation into the local adaptation of *Kandelia obovata* populations across China provides critical insights into the genetic dynamics and adaptive evolution of mangroves under global climate change. Through whole-genome resequencing analysis, we uncovered a strong population structure with complex demographic events such as expansion, bottlenecks, and gene flow, highlighting the intricate historical context within which local adaptation has occurred. Notably, genetic differentiation is high among the geographically close *K. obovata* populations, likely due to limited gene flow as a result of human activities. Genome-wide scans of population differentiation pinpointed regions under selective sweeps, with more intense signals in northern populations. Our findings emphasize the importance of temperature in driving genetic adaptations, as opposed to precipitation, which showed no discernible genotype-environment associations. The southmost Wenchang population exhibited minimal selective sweep signatures, reflecting the low selective pressure in this tropic region, potentially confounded by gene flow from other populations. We identified 39 candidate genes with high confidence responsible for local adaptation, enriched in stimulus response functions and largely different from those genes involved in the speciation of *K. obovata* from *K. candel*, which are associated with basic cellular functions. These findings set the stage for further research to explore the molecular basis of local adaptation and resilience of mangroves to environmental stress. Such knowledge is vital for conservation strategies and predictive modeling of species responses in an era of rapid climate change.

## Data availability statement

The datasets presented in this study can be found in online repositories. The names of the repository/repositories and accession number(s) can be found in the article/**Supplementary Material**.

## Author contributions

CZ: Writing – review & editing, Writing – original draft, Visualization, Software, Methodology, Investigation, Formal analysis, Data curation. YW: Writing – review & editing, Software, Methodology, Investigation, Formal analysis. RZ: Writing – review & editing, Supervision, Resources, Funding acquisition, Conceptualization. TT: Writing – review & editing, Writing – original draft, Validation, Supervision, Project administration, Funding acquisition, Conceptualization.
