# Supplementary material for: Genetic basis of local adaptation in the cold-tolerant mangrove Kandelia obovata
Source: Front Plant Sci. 2024 Apr 24;15:1385210. doi: 10.3389/fpls.2024.1385210 (PMC11076828; doi:10.3389/fpls.2024.1385210)
Supplement: Supplementary file 1 [file DataSheet_1.zip › Figures S1 - S4.DOCX]

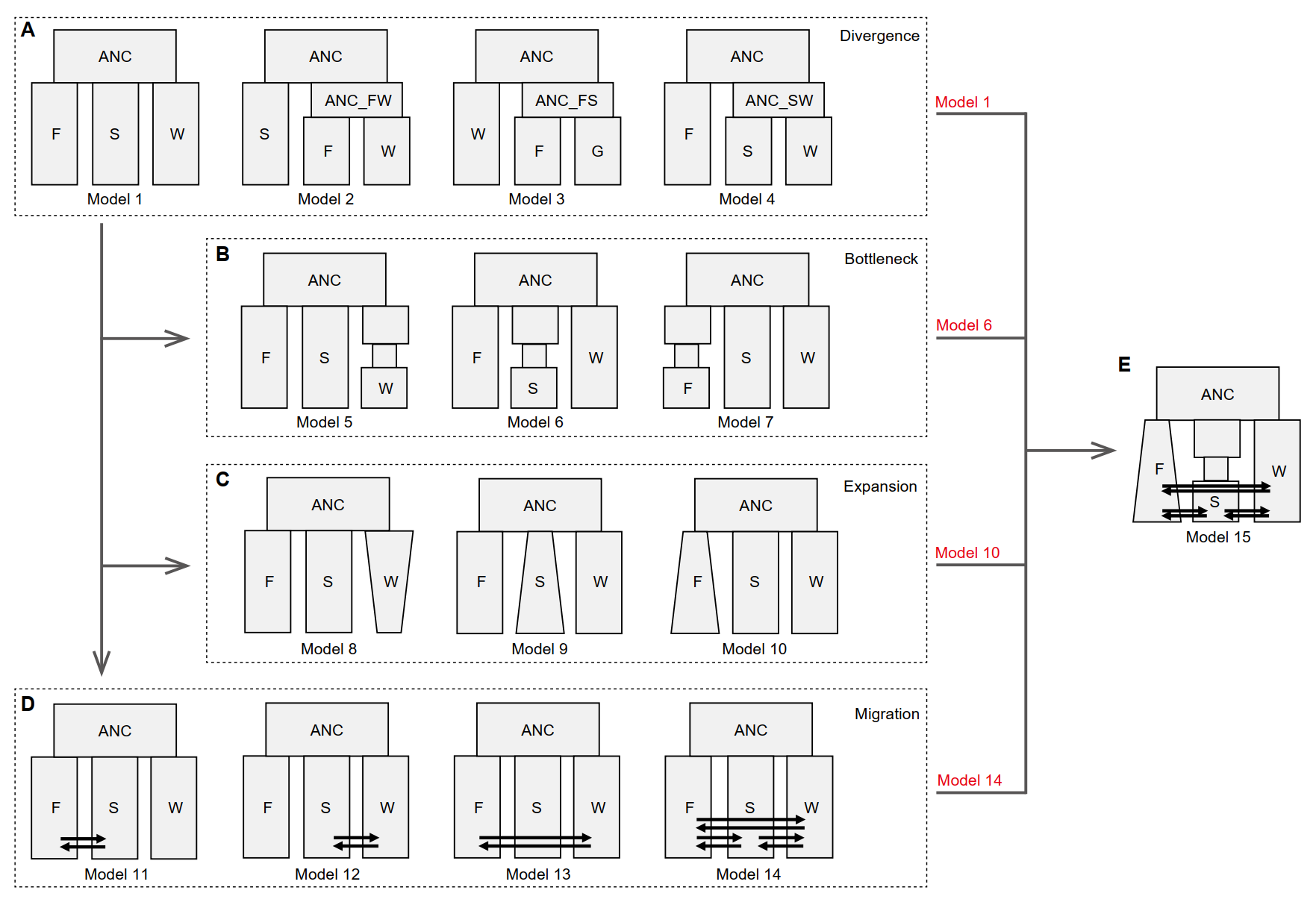


**Supplementary FIGURE 1 Demographic models tested in this study. (A)** Models of divergence group. Model 1: depicts the scenario where three populations diverged from a single ancestral population (ANC) without further interaction. Model 2-4: Represent two-step divergence scenarios, where the three populations diverged in a sequential manner rather than all at once. **(B)** Models of bottleneck group. Model 5-7: These are variations of Model 1, where following the initial isolation, one of the three populations undergoes a bottleneck, which involves population size reduction followed by recovery. **(C)** Models of expansion group. Model 8-10: Theses models expand on model 1 by proposing that following the initial isolation, one of the three populations experiences exponential growth in population size. **(D)** Models of migration group. Model 11-14: Based on the initial isolation in Model 1, these models introduce asymmetric gene flow (migration) between populations. The asymmetry refers to different rates or timings of gene flow among the populations. **(E)** Combined Model. Model 15: This model synthesizes elements from the highest Akaike’s weight values models (as per Table S3aa-d) from previous four groups. It assumes isolation of the three populations with asymmetric gene flow, where the Fuding population undergoes exponential population size change, and the Shenzhen population experiences a bottleneck.

**
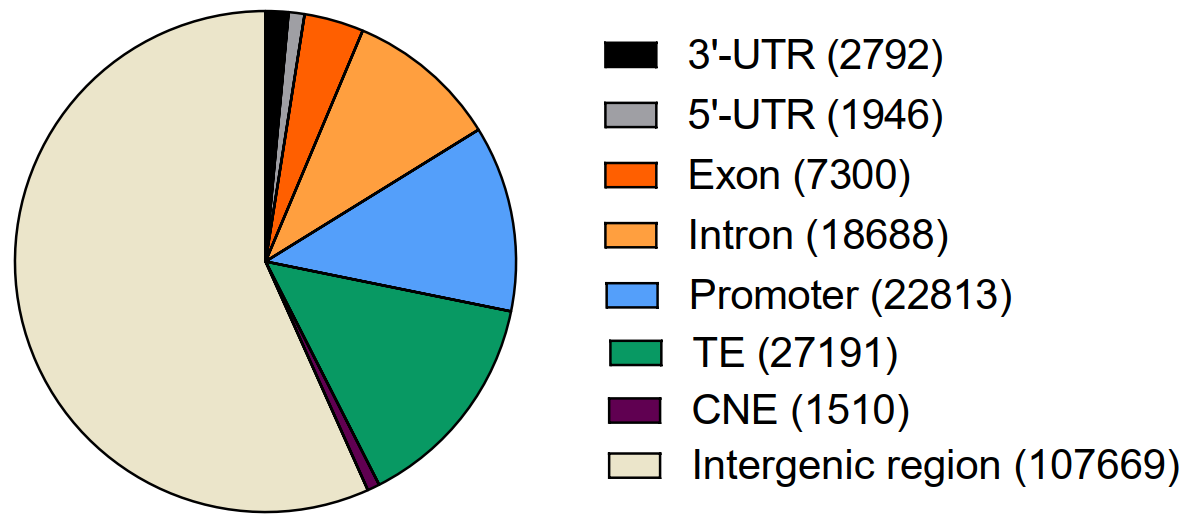
**

**Supplementary FIGURE 2 Distribution of SNPs in different genomic features.** 3’UTR, 3′ untranslated region. 5’UTR, 5′ untranslated region. TE, transposable elements. CNE, conserved non-coding elements.


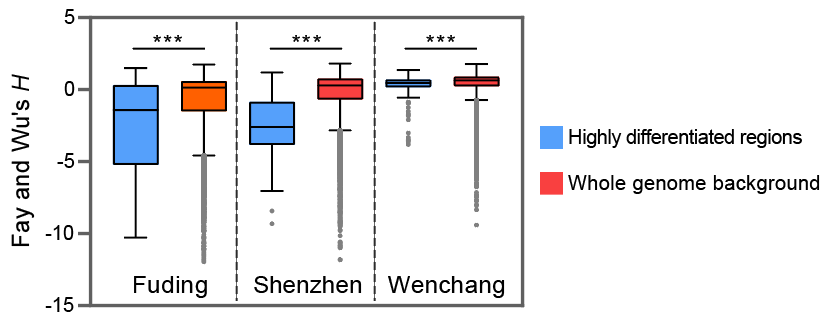


**Supplementary FIGURE 3 Distribution of Fay and Wu’s *H* statistics in highly differentiated regions (HDRs) versus the entire genome.** This boxplot illustrates the variation Fay and Wu’s *H* values within HDRs (depicted in blue) in comparison with the whole-genome background (depicted in red). A sliding window approach was adopted, applying 2-kb windows with 5-kp steps, using SNPs identified in HDRs or across the entire *K. obovata* genome for each of the three studied populations, separately. Asterisks indicate the significance level of Mann-Whitney U test: ***, *p*-value < 0.001.


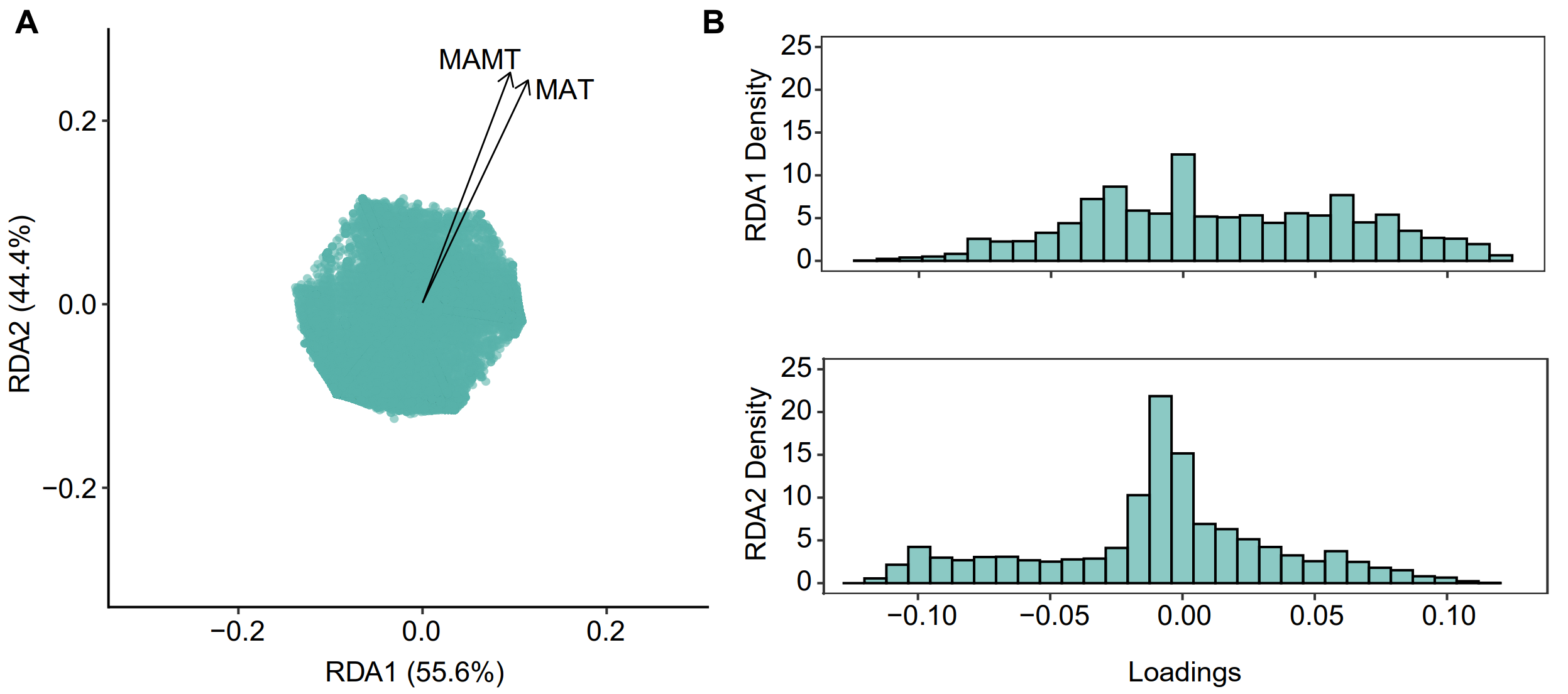


**Supplementary FIGURE 4 Redundancy analysis (RDA). (A)** RDA testing the effect of environmental variables, including mean annual temperature (MAT) and mean annual minimum temperature (MAMT), on the degree of genetic differentiation. The plot displays the first two canonical axes, RDA1 (55.6%) and RDA2 (44.4%). **(B)** Distributions of SNP loadings on the first RDA axis and the second RDA axis among populations.
